# Risk Factors for Long-Term Death After Medullary Infarction: A Multicenter Follow-Up Study

**DOI:** 10.3389/fneur.2021.615230

**Published:** 2021-03-04

**Authors:** Dao Pei Zhang, Xiang Zhe Liu, Suo Yin, Qian Kun Ma, Meng Yu, Huai Liang Zhang, Xin Zhi Wang, Jie Wen Zhang

**Affiliations:** ^1^Department of Neurology, The First Affiliated Hospital of Henan University of CM, Zhengzhou, China; ^2^Department of Image, People's Hospital of Henan University of CM, Zhengzhou, China; ^3^Department of Image, People's Hospital of Zhengzhou University, Zhengzhou, China; ^4^Department of Neurology, People's Hospital of Henan University of CM, Zhengzhou, China; ^5^Department of Image, The First Affiliated Hospital of Henan University of CM, Zhengzhou, China; ^6^Department of Neurolgoy, People's Hospital of Zhengzhou University, Zhengzhou, China

**Keywords:** medullary infarction, prognosis, death, risk factors, stroke

## Abstract

**Background and Purpose:** We investigated the risk factors for death in patients with medullary infarction (MI) during a long-term follow-up.

**Methods:** We retrospectively examined 179 consecutive patients (130 men and 49 women) who had clinical and MRI findings consistent with MI between February 2012 and January 2017 at three university hospitals. Long-term outcomes were assessed by telephonic interview. The clinical and radiological features and risk factors for poor outcomes (modified Rankin scale score ≥ 3, all-cause death) were analyzed.

**Results:** Mean age of patients was 58.3 ± 12.8 years (range, 25–87); mean follow-up period after stroke onset was 42.7 ± 13.2 months (range, 24–78). Basilar artery (BA) stenosis >50% was more closely related to medial medullary infarction (MMI) than other types. There was greater frequency of ipsilateral vertebral artery hypoplasia (VAH) or V4AH and V4 occlusion in lateral MI than in other types. On rostro-caudal classification, middle (M)+dorsal (D) was most frequent, followed by the ventral (V)+M+D types. 21.2% patients showed poor long-term prognosis. Age ≥ 65 years, recurrent stroke, dysphagia, >50% BA stenosis, and ventral MI were risk factors for poor long-term prognosis. All-cause mortality rate was 10.6%; age ≥ 65 years, recurrent stroke, and dysphagia were risk factors for death in the long-term. Ventral MI and MMI+cerebellar infarction, as well as stroke mechanism of artery-to-artery embolism, were potential risk factors for death in the long-term. Pneumonia and recurrent stroke were major causes of death.

**Conclusions:** Long-term poor outcomes of MI and all-cause mortality were not infrequent. Older age, recurrent stroke, and dysphagia were common risk factors for poor prognosis and death.

## Introduction

Medullary infarction (MI) is a rare clinical entity that can be classified into lateral and medial medullary infarction (LMI and MMI) based on the clinical and lesion patterns (1). Numerous studies have characterized the clinical manifestations of MI and their association with the anatomical site of MI (2–4). In some previous small-scale studies, most patients with MI were found to have a favorable prognosis with a low rate of mortality or clinical progression (5, 6). The lesion patterns of MI are heterogeneous owing to the unique arterial supply of medulla; therefore, there is considerable variability in the clinical manifestations of MI. Moreover, the correlation of clinical manifestations with stroke etiology, mechanism, and the prognosis of these patients is not well-characterized (7–9).

In particular, the long-term prognosis of these patients has seldom been discussed. Furthermore, there is a paucity of studies that have investigated the risk factors for survival and death in MI patients on long-term follow-up (1). Therefore, we examined the clinical and imaging features and etiopathogenesis and analyzed the risk factors for death during long-term follow-up of MI patients treated at three tertiary hospitals.

## Patients and Methods

### Study Patients

We retrospectively examined 245 consecutive patients who had clinical and MRI imaging findings consistent with MI at three tertiary hospitals (People's Hospital of Zhengzhou University, the First Affiliated Hospital, and People's Hospital of Henan University of Chinese Medicine) between February 2012 and January 2017. The imaging records were retrieved from the Picture Archiving and Communication Systems (PCAS) at the three hospitals. The exclusion criteria were: patients who died in the acute phase (*n* = 4); patients who were admitted >7 days after symptom onset (*n* = 9), patients with concomitant major infarction outside the medulla including pontine, midbrain, and occipital or thalamic infarction (*n* = 21); and patients who had significant neurological sequelae attributable to previous stroke (*n* = 7). However, we included patients who showed diffusion-weighted MRI (DWI)-identified lesions (no space occupying effect) in the cerebellum (*n* = 28), because these patients may have lesions of the ispilateral vertebral artery or posterior inferior cerebellar artery in common, and there was a patient with LMI+MMI+ cerebellar infarction (C) who was classified in the LMI+MMI group. Twenty-five patients could not be followed-up because of the inability to contact them by telephone (*n* = 16) or their refusal to participate (*n* = 9). Thus, 179 patients with acute MI were included in the analysis. Data pertaining to all patients were examined by the first author, and the specific signs and symptoms recorded.

The definition of risk factors, such as hypertension, diabetes mellitus, hyperlipidemia, coronary heart disease, atrial fibrillation, smoking, alcohol intake, and stroke history work-up protocol, at our institutes are described elsewhere (10).

Written informed consent was obtained from all participants or their legal surrogates.

### Imaging Analysis

We performed DWI of acute patients and then follow-up fluid-attenuated inversion recovery/T2/T1-weighted MRI and magnetic resonance angiography (MRA) of patients with acute (<3 days after onset) stroke; in addition, some patients who had sustained focal neurological defect underwent follow-up DWI to exclude false negative results of early DWI. In patients who were admitted >3 days after stroke onset, MRI and MRA were performed only once. MRI examinations were performed using either a 1.5 Tesla or 3.0 Tesla MR imaging unit (GE Medical, Piscataway, NJ, USA). A horizontal plane at 3-mm intervals from the medulla to the midbrain was obtained. The DWI parameters were: repetition time (TR), 7,500 ms; echo time (TE), 84 ms; matrix number, 128 × 128; and two *b*-values of 0 and 1,000 s/mm. Three-dimensional (3D)-time-of-flight (TOF)-MRA and 3D-contrast-enhanced (CE)-MRA were also performed at the time of MR imaging with parameters described elsewhere (10).

### Evaluation of Arterial Stenoses

The degree of arterial stenoses was categorized as mild (≤ 50% diameter reduction), moderate or severe (>50% diameter reduction with complete distal flow), occlusion, and aplasia [non-visualization of the entire vertebral artery (VA)]/hypoplasia [diffuse homogeneous narrowing of the entire basilar artery (BA) or VA]. We interpreted non-visualization or homogeneous narrowing of the distal VA after the origin of the posterior inferior cerebellar artery as aplasia/hypoplasia (V4AH), and irregular narrowing as atherosclerotic vascular stenosis. MRA showing double lumen, intimal flap, pearl and string sign, or improvement of vascular stenosis of the VAs or proximal BA within several days were classified as probable dissection (11). The diameter of the VA was measured at three consecutive points from the bilateral VA junction (3 mm apart) and only the maximum value was considered. VAH was presumed when VA met the following morphologic criteria on MRA: VA diameter <2.5 mm and a concomitant diameter asymmetry ratio of <1:1.7 throughout the VA (12). BA diameter was measured at the mid-pons level on TOF source images. In the present study, vertebrobasilar dolichoectasia (VBD) was defined as BA diameter > 4.5 mm or VA diameter > 4.0 mm (10).

### Assessment of Lesion Pattern and Etiology

#### Distribution of Infarcts Based on MRI Findings

As lesions were often invisible or vague on the initial DWI in some patients, those who had sustained a focal neurological defect underwent repeat imaging evaluation (33 patients). We used the MRI (either DWI or T2) findings obtained in the subacute stage (mean, 3.1 ± 1.2 days after stroke onset). Lesions were classified by two authors (YS and ZDP) who were blinded to clinical information; in case of any disagreement between the two, a topographical consensus was achieved by participation of a third neurologist. Rostro-caudally, the lesions were categorized as “rostral,” “middle,” and “caudal,” according to criteria described previously (4). The lesions of medulla were ventro-dorsally classified according to the diagram of the rostral, middle, and caudal medulla as (4) “ventral (V)” (ventral part, presumably containing the pyramid); “middle (M)” (middle part, presumably including the medial lemniscus); and “dorsal (D)” (dorsal part, presumably including the medial longitudinal fasciculus [MLF] in a lesion extending to the dorsal surface of the medulla). According to lesions located in the whole medulla, MI patients were divided into LMI, MMI, bilateral MMI (BMMI), and hemi-medullary infarction (HMI), LMI+MMI, LMI+C, and MMI+C.

#### Presumed Stroke Mechanisms

The presumed mechanism of stroke was categorized by consensus among our stroke team with modification of recent guidelines and as described in a previous major study (3, 10, 13).

**Figure 1:**
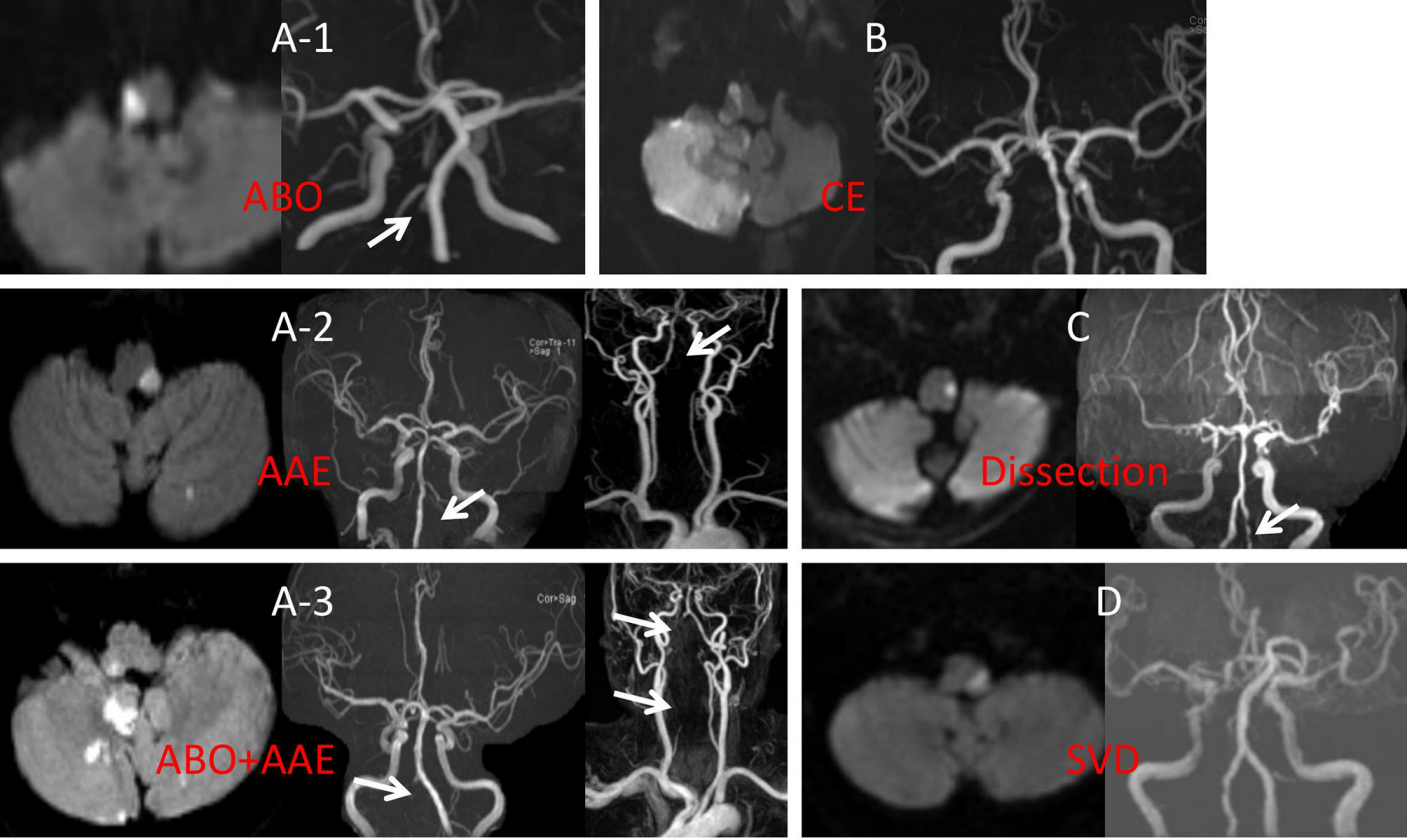
**(A-1)** DWI showed right caudal medullary infarction, MRA showed right intracranial VA occlusion. **(A-2)** DWI indicated left middle medullary and cerebellar infarction, MRA showed occlusion in the proximal left VA with no distal VA disease. **(A-3)** DWI showed right rostral medullary and cerebellar multiple infarction, MRA showed in both distal and proximal right VA. **(B)** DWI showed the right rostral medullary and cerebellar multiple infarction, MRA showed bilateral VA was normal. **(C)** DWI showed caudal medullary infarction, MRA showed the pearl, and string sign of left VA. **(D)** DWI showed middle medullary infarction, MRA showed the bilateral VA were normal. ABO, atheromatous branch occlusion; AAE, artery-to-artery embolism; CE, cardiogenic embolism; SVD, small vessel disease; DWI, diffusion weighted imaging; MRA, magnetic resonance angiography; VA, vertebral artery.

1. Large vessel disease (LVD). LVD was divided into three categories: (1) atheromatous branch occlusion (ABO); (2) artery-to-artery embolism (AAE); and (3) AAE+ABO. 2. Cardiogenic embolism (CE). 3. VA dissection (DIS). 4. Small vessel disease (SVD). 5. Undetermined (UN) etiology (Figures 1A–D).

### Follow-Up Study

In patients with a follow-up period > 24 months, telephonic interviews were conducted in February 2019 by an experienced stroke research coordinator (Y.S.) who certified the modified Rankin scale (mRS) score using a structured format, exploring the general neurological outcome using a modified Rankin scale (0–5). mRS scores ≥ 3 were considered as poor outcome and all-cause mortality was elaborately recorded. The subjective sensory complaints of patients were assessed as described previously (3). The severity was assessed using 10-point markers on a visual numeric scale (1, slight; 10, most severe). Dysphagia was recorded by reviewing medical records at admission (these patients were screened by drinking water test in Wadi) and patients' complaints during follow-up. The cause of death and incidence of recurrent stroke were confirmed by telephonic interviews and medical records.

### Statistical Analysis

We used the Chi-squared test to compare categorical variables; the Fisher exact test was used when the number of cells was small. The *t*-test was used to compare continuous variables. Potential risk factors (*P* < 0.20) identified on univariate analysis were included in the multivariate analysis after adjusting for age and sex. Statistical tests were performed with a 2-tailed α level of 0.05. Data were analyzed with IBM SPSS version 13.0.

## Results

### Demographic and Clinical Features

The study population comprised of 130 men and 49 women; the mean age of the patients was 58.3 ± 12.8 years (range, 25–87). Risk factors included hypertension in 113 (63.1%) patients, diabetes mellitus in 65 (36.3%), smoking in 72 (40.2%), alcohol consumption in 56 (31.3%), hyperlipidemia in 39 (21.8%), and atrial fibrillation in 8 (4.5%) patients. Thirty-seven (20.7%) patients had a history of stroke, and 27 (15.1%) patients had a history of coronary heart disease (Table 1).

**Table 1:** Clinical characteristics of 179 patients with medullary infarction.

	**LMI** **(*n* = 96)**	**MMI** **(*n* = 42)**	**BMMI** **(*n* = 9)**	**LMI+MMI** **(*n* = 1)**	**HMI** **(*n* = 3)**	**LMI+C** **(*n* = 23)**	**MMI+C** **(*n* = 5)**
Age	58.8 ± 12.5	58.9 ± 14.6	57.9 ± 10.2	72.0	53.7 ± 7.6	56.3 ± 12.7	64.6 ± 16.1
Sex(male)	67 (69.8)	31 (73.8)	6 (66.7)	1 (100.0)	3 (100.0)	21 (91.3)	2 (40.0)
**Paralysis**							
No	34 (35.4)	15 (35.7)	7 (77.8)	0	0	12 (52.2)	2 (40.0)
Monoplegia	8 (8.3)	5 (11.9)	1 (11.1)	0	0	2 (8.7)	0
Hemiplegia	42 (43.8)	19 (45.2)	1 (11.1)	1 (100.0)	2 (66.7)	4 (17.4)	2 (40.0)
Quadriplegia	12 (12.5)	3 (7.1)	0	0	1 (33.3)	5 (21.7)	1 (20.0)
**Paresthesia**							
No	10 (10.4)	2 (4.8)	4 (44.4)	0	1 (33.3)	6 (26.1)	3 (60.0)
Numb/hypalgesia	37 (38.5)	17 (40.5)	3 (33.3)	0	2 (66.7)	7 (30.4)	1 (20.0)
Others	49 (51.0)	23 (54.8)	2 (22.2)	1 (100.0)	0	10 (43.5)	1 (20.0)
Vertigo/dizziness	77 (80.2)	34 (81.0)	7 (77.8)	1 (100.0)	2 (66.7)	19 (82.6)	1 (20.0)
Dysphagia	27 (28.1)	12 (28.6)	5 (55.6)	0	2 (66.7)	9 (39.1)	1 (20.0)
Dysarthria	34 (41.5)	16 (38.1)	5 (55.6)	0	2 (66.7)	10 (43.5)	1 (20.0)
Facial paralysis	13 (13.5)	10 (23.8)	2 (22.2)	0	1 (33.3)	10 (43.5)	1 (20.0)
Nausea/vomiting	38 (39.6)	24 (57.1)	5 (55.6)	1 (100.0)	2 (66.7)	11 (47.8)	2 (40.0)
Ataxia	29 (30.2)	7 (16.7)	3 (33.3)	0	2 (66.7)	18 (78.3)	0
Nystagmus	13 (13.5)	5 (11.9)	1 (11.1)	1 (100.0)	1 (33.3)	6 (26.1)	0
Diplopia	8 (8.3)	1 (2.4)	0	1 (100.0)	0	2 (8.7)	0
Headache	21 (21.9)	14 (33.3)	3 (33.3)	0	2 (66.7)	2 (8.7)	1 (20.0)
Horner signs	5 (5.2)	3 (7.1)	2 (22.2)	1 (100.0)	1 (33.3)	2 (8.7)	0
**Medical history**							
Hypertension	63 (65.6)	24 (57.1)	4 (44.4)	1 (100.0)	1 (33.3)	17 (73.9)	3 (60.0)
Diabetes mellitus	37 (38.5)	14 (33.3)	2 (22.2)	0	1 (33.3)	9 (39.1)	2 (40.0)
Atrial fibrillation	1 (1.0)	2 (4.8)	0	0	0	5 (21.7)	0
Coronary heart disease	13 (13.5)	6 (14.3)	1 (11.1)	0	1 (33.3)	5 (21.7)	1 (20.0)
Hyperlipidemia	22 (22.9)	9 (21.4)	1 (11.1)	0	1 (33.3)	6 (26.1)	0
Strokes	17 (17.7)	7 (16.7)	1 (11.1)	1 (100.0)	1 (33.3)	9 (39.1)	2 (40.0)
Drinking	26 (27.1)	13 (31.0)	4 (44.4)	0	0	13 (56.5)	0
Smoking	39 (40.6)	18 (42.9)	3 (33.3)	0	0	11 (47.8)	0
**Long-term follow up**							
mRS ≥ 3	17 (17.7)	10 (23.8)	3 (33.3)	1 (100.0)	3 (100.0)	5 (21.7)	1 (20.0)
Death	7 (7.3)	7 (16.7)	0	0	0	3 (13.0)	2 (40.0)
Recurrent stroke	4 (4.2)	4 (9.5)	0	0	0	3 (13.0)	0

*LMI, lateral medullary infarction; MMI, medial medullary infarction; LMI+C, LMI+cerebellar infarction; MMI+C, MMI+cerebellar infarction; BMMI, bilateral MMI; HMI, hemimedullary infarction; mRS, modified Rankin Scale*.

The most common syndromes were LMI in 96 patients (53.6%), MMI in 42 (23.5%), LMI+C in 23 (12.8%), BMMI in nine (5.0%), MMI+C in five (2.8%), HMI (infarction area > 40% of the medulla in one place) in three (1.7%), and LMI+MMI in one (0.6%) patient.

Sensory impairment was the most common symptom (154 patients); however, it could not be reliably assessed in nine patients because of severe dysarthria or confusion. Among the remaining 145 patients, sensory symptoms/signs were observed in 118 patients (81.3%). Motor dysfunction was the second most common symptom (107 patients): hemiparesis in 70, quadriparesis in 20, and monoparesis in 17 patients. The motor dysfunction was severe (medical research council scale < 3 in any proximal limb) in 39 patients (36.4%). Thirty-seven patients showed mild facial paresis on the ipsilateral side (Table 1).

Limb ataxia was noticed in 84 patients, usually associated with mild weakness (ataxic hemiparesis). Dysarthria was present in 55 patients. Dysphagia was noted in 68 patients, 16 of whom required a nasogastric tube for feeding. The other symptoms were vertigo/dizziness (*n* = 82), headache (*n* = 43), Horner syndrome (*n* = 13), nausea/vomiting (*n* = 12), and diplopia (*n* = 12). Twenty-seven patients had nystagmus. Six patients showed internuclear ophthalmoplegia (Table 1).

### MRI Findings and Presumed Mechanism of Stroke

Initial DWI imaging was negative in 33 (18.4%) patients. These lesions represented 35.7% of MMI, 17.7% of LMI (χ^2^ = 7.248, *P* = 0.048), and 20.0% of MMI+C. Of these, 42.4% were in the rostral, 26.7% were in the middle, and 30.9% were in the caudal medullary region. Risk factors and etiological mechanisms were not significantly different between patients with and without visible infarction on the initial DWI.

As shown in Table 2, vertebrobasliar artery lesions included BA stenosis >50% in 31 (17.3%), ipsilateral VA stenosis >50% in 62 (34.6%), ipsilateral V4 occlusion in 22 (12.3%), VBD in 32 (17.9%), basilar artery hypoplasia in nine (5.0%), and ipsilateral VAH or V4AH in 76 (42.5%) patients. Forty-seven patients had ABO (26.3%), 40 had AAE (22.3%), and 19 had ABO+AAE (10.6%). Eighteen patients were considered to have SVD (10.1%). Eight were categorized as CE (4.5%). Twenty patients had VA dissection (11.2%). The etiology was unknown in 23 (12.8%) patients. There was no significant difference with respect to stroke mechanism among LMI, MMI, BMMI, HMI, LMI+C, and MMI+C (Table 2).

**Table 2:** Imaging characteristics of 179 patients with medullary infarction.

	**LMI** **(*n* = 96)**	**MMI** **(*n* = 42)**	**BMMI** **(*n* = 9)**	**LMI+MMI** **(*n* = 1)**	**HMI** **(*n* = 3)**	**LMI+C** **(*n* = 23)**	**MMI+C** **(*n* = 5)**
**MRA**							
BA stenosis > 50%	13 (13.5)	7 (16.7)	5 (55.6)	0	1 (33.3)	3 (13.0)	2 (40.0)
VA stenosis > 50%	32 (33.3)	16 (38.1)	2 (22.2)	0	0	9 (39.1)	3 (60.0)
V4 occlusion	12 (12.5)	5 (11.9)	0	1 (100.0)	0	4 (17.4)	0
VBD	18 (18.8)	7 (16.7)	2 (22.2)	0	0	5 (21.7)	0
BAH	2 (2.1)	4 (9.5)	3 (33.3)	0	0	0	0
VAH or V4AH	48 (50.0)	18 (42.9)	1 (11.1)	1 (100.0)	3 (100.0)	12 (52.2)	1 (20.0)
**Lesions location**							
Rostral	18 (18.8)	28 (66.7)	4 (44.4)	1 (100.0)	0	9 (39.1)	2 (40.0)
Middle	48 (50.0)	5 (11.9)	2 (22.2)	0	2 (66.7)	7 (30.4)	1 (20.0)
Caudal	10 (10.4)	3 (7.1)	0	0	0	2 (8.7)	0
R+M	9 (9.4)	5 (11.9)	2 (22.2)	0	1 (33.3)	3 (13.0)	2 (40.0)
M+C	11 (11.5)	2 (4.8)	1 (11.1)	0	0	1 (4.3)	0
Ventral	2 (2.1)	26 (61.9)	2 (22.2)	0	0	1 (4.3)	1 (20)
Middle	30 (31.3)	4 (9.5)	0	0	0	8 (34.8)	0
Dorsal	45 (46.9)	1 (2.4)	0	0	0	12 (52.2)	0
V+M	0	1 (2.4)	0	0	0	0	0
M+D	12 (12.5)	3 (7.1)	0	0	0	2 (8.7)	2 (40.0)
V+D	0	0	1 (11.1)	0	0	0	0
V+M+D	1 (1.0)	6 (14.3)	5 (55.6)	1 (100.0)	3 (100.0)	0	1 (20.0)
**Stroke mechanisms**							
ABO	27 (28.1)	12 (28.6)	1 (11.1)	0	1 (33.3)	6 (26.1)	0
AAE	24 (25.0)	8 (19.0)	2 (22.2)	0	1 (33.3)	2 (8.7)	2 (40.0)
ABO+AAE	5 (5.2)	2 (4.8)	5 (55.6)	0	1 (33.3)	5 (21.7)	1 (20.0)
SVD	13 (13.5)	4 (9.5)	0	0	0	0	0
DIS	10 (10.4)	5 (11.9)	2 (22.2)	1 (100.)	0	2 (8.7)	0
CE	1 (1.0)	2 (4.8)	0	0	0	4 (17.4)	2 (40.0)
UN	14 (14.6)	9 (21.4)	0	0	0	0	0
**Initial DWI negative**	17 (17.7)	15 (35.7)	0	0	0	0	1 (20.0)

*ABO, atheromatous branch occlusion; AAE, artery-to-artery embolism; CE, cardiogenic embolism; SVD, small vessel disease; UN, undetermined etiology; DIS, dissection; LMI, lateral medullary infarction; MMI, medial medullary infarction; LMI+C, LMI+cerebellar infarction; MMI+C, MMI+cerebellar infarction; BMMI, bilateral MMI; HMI, hemimedullary infarction; VA, vertebral artery; BA, basilar artery; VAH, vertebral artery hypoplasia; BAH, basilar artery hypoplasia; VBD, vertebrobasilar dolichoectasia; R, rostral; M, middle; C, caudal; V, ventral; D, dorsal; DWI, diffusion weighted imaging*.

**Figure 2:**
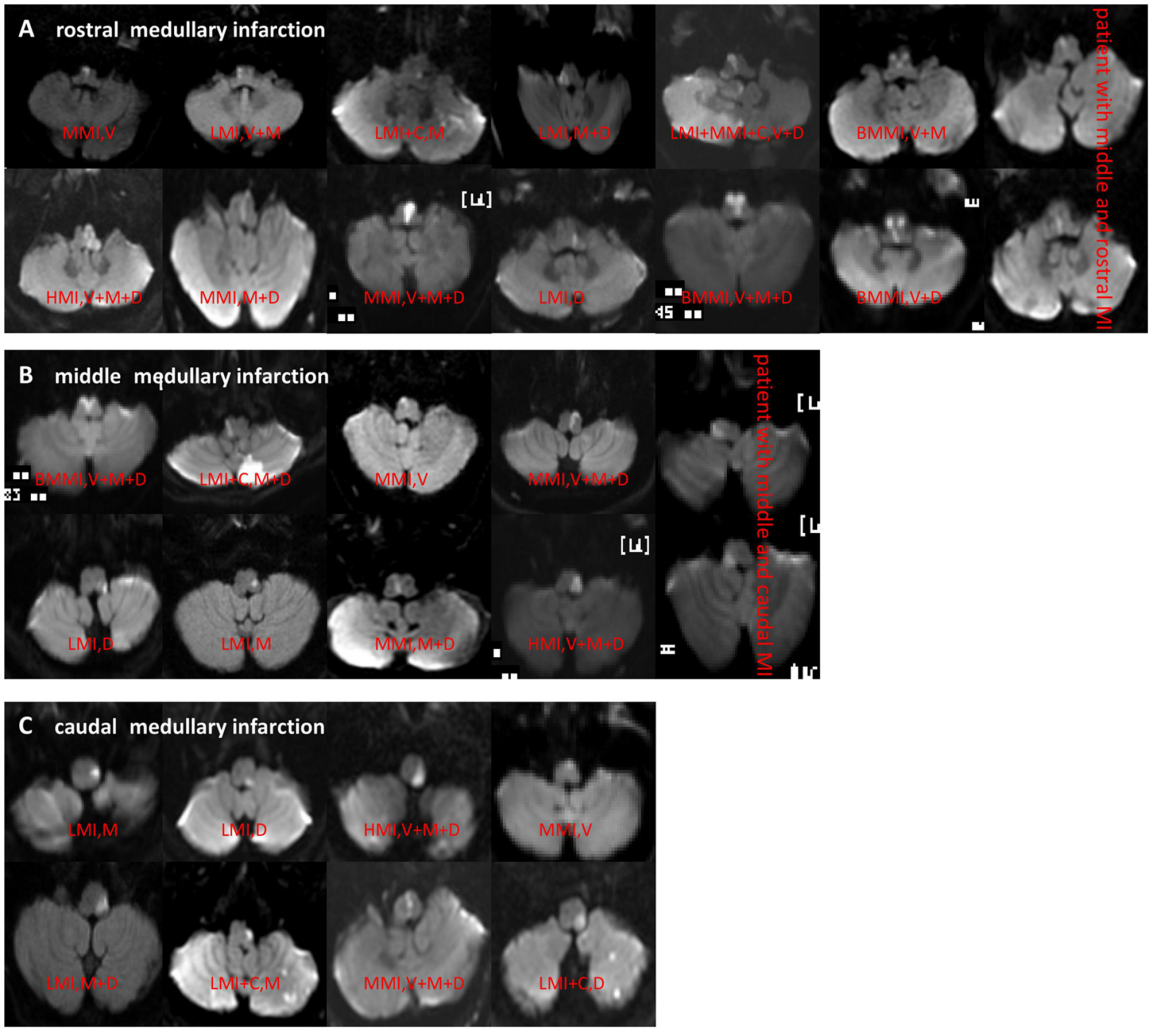
**(A–C)** DWI showed different types of rostral, middle, and caudal MI. MI, medullary infarction; LMI, lateral MI; MMI, medial MI; BMMI, bilateral MMI; LMI+C, LMI+cerebellar infarction; MMI+C, MMI+cerebellar infarction; HMI, hemimedullary infarction; V, ventral; M, middle; D, dorsal.

On rostro-caudal classification, the lesions were located in the middle medulla (*n* = 65), rostral (*n* = 62), middle+rostral (*n* = 22), caudal (*n* = 15), and middle+caudal (*n* = 15). Ventro-dorsally, the lesions were located in the ventral medulla (*n* = 32), middle (*n* = 42), dorsal (*n* = 58), M+D (*n* = 19), V+M+D (*n* = 17), V+M (*n* = 3), and V+D (*n* = 1). M+D type was the most frequent, followed by the V+M+D type (Table 2; Figures 2A–C).

BA stenosis >50% was more closely related to MMI than the others (χ^2^ = 9.749, *P* = 0.008). LMI mostly occurred in the middle medulla, and MMI mostly occurred in the rostral medulla. The ventral medulla was the most frequently affected in MMI, while the dorsal medulla was the most frequently affected in LMI. The LMI group had a significantly greater proportion of patients with occlusion of ipsilateral VAH or V4AH (χ^2^ = 5.989, *P* = 0.049) and V4 (χ^2^ = 10.595, *P* < 0.001) than in the other groups (Table 2).

### Risk Factors for Poor Prognosis and Death During Long-Term Follow-Up

Telephonic interviews of 179 patients were conducted 24–78 months (mean 42.7 ± 13.2 months) after stroke onset. During follow-up, 19 patients were found to have died; of these, six died of pneumonia (2, 3, 3, 4, 4, and 6 years post-stroke, respectively), four due to recurrent stroke (3, 5, 5, and 6 years post-stroke, respectively), three due to myocardial infarction (2, 3, and 6 years post-stroke, respectively), one due to traumatic brain hemorrhage (3 years post-stroke), one due to lung cancer (2 years post-stroke), and one due to cerebral hemorrhage (6 years post-stroke). The cause of death of three patients was uncertain. Non-fatal stroke and recurrent stroke occurred in 11 patients (6.1%; Table 3).

**Table 3:** Characteristics of 19 dead patients with medullary infarction.

**Patient**	**Age/sex**	**Risk factors**	**Stroke mechanisms**	**Major symptoms and signs**	**Lesions location of medulla**	**Magnetic resonance angiography**	**Death time**	**Causes of death**
1	83/M	No	UN	Vertigo, headache, vomit, hemiplegia, dysphasia	MMI, rostral, ventral	V4AH	2 years	Pneumonia
2	47/M	DM, CHD, HT, stroke, hyperlipemia	ABO	Hemiplegia, dysphasia	MMI, middle-caudal, V+M+D	VA stenosis	6 years	Pneumonia
3	62/M	HT, smoking	ABO	Vertigo, hemidysesthesia, hemiplegia, dysphasia	MMI, rostral, ventral	BA and VA stenosis	4 years	Pneumonia
4	52/M	HT, DM	UN	Vertigo, vomit, diplopia, hemidysesthesia, hemiplegia, dysphasia	LMI, middle, dorsal	V4AH	3 years	Myocardial infarction
5	70/F	HT	AAE	Vertigo, hemiplegia	MMI+C, middle, M+D	VA stenosis	2 years	Lung cancer
6	70/M	HT, DM, smoking, stroke	UN	Vertigo, ataxia, dysarthria, hemidysesthesia, hemiplegia, dysphasia	LMI, caudal, middle	VBD	6 years	Myocardial infarction
7	69/M	HT, DM, CHD, stroke, hyperlipemia	AAE	Vertigo, facial paralysis, dysarthria hemidysesthesia, nystagmus, hemiplegia, dysphasia	LMI+C, R+M, dorsal	VBD	3 years	Traumatic brain hemorrhage
8	78/M	smoking, drinking	ABO+AAE	Vertigo, ataxia, nystagmus, hemidysesthesia	LMI+C, middle, dorsal	VA stenosis	5 years	Recurrent stroke
9	76/F	HT, DM	DIS	Hemidysesthesia, hemiplegia	LMI, middle, ventral	VA string	4 years	Pneumonia
10	78/M	CHD, stroke, smoking, drinking	UN	Vertigo, vomit, monoplegia, dysarthria, hemidysesthesia, nystagmus	LMI, R+M, dorsal	V4AH	3 years	Recurrent stroke
11	59/M	DM	AAE	Vertigo, hemiplegia, dysphasia	MMI, R+M, ventral	VBD, VAstenosis	6 years	Recurrent stroke
12	87/M	HT, atrial fibrillation, stroke	CE	Dysarthria, facial paralysis, quadriplegia, dysphasia	MMI+C, rostral, ventral	VA stenosis	2 years	Uncertainty
13	75/F	HT, CHD, stroke, hyperlipemia	ABO+AAE	Vertigo, vomit, dysarthria hemidysesthesia, nystagmus, dysphasia	LMI, R+M, M+D	VA stenosis	3 years	Pneumonia
14	71/F	hyperlipemia	UN	Vertigo, dysarthria, nystagmus, ataxia, dysphasia	LMI, M+C, middle	V4AH	5 years	Recurrent stroke
15	68/F	HT, CHD	AAE	Vertigo, facial paralysis, ataxia hemidysesthesia, nystagmus, hemiplegia, dysphasia	MMI, middle, ventral	VA stenosis	6 years	Brain hemorrhage
16	58/F	HT, CHD, atrial fibrillation	CE	Vertigo, facial paralysis, ataxia hemidysesthesia, nystagmus, hemiplegia	MMI, rostral, V+M+D	BA stenosis	3 years	Uncertainty
17	80/F	smoking	UN	Vertigo, vomit, headache, facial paralysis, dysphasia, hemidysesthesia, nystagmus hemiplegia	MMI, middle, M+D	VA stenosis, VAH	2 years	Myocardial infarction
18	83/M	HT, stroke	SVD	Vertigo, hemidysesthesia, hemiplegia, ataxia	LMI, rostral, dorsal	normal	2 years	Uncertainty
19	76/F	HT, DM, CHD, atrial fibrillation	CE	Vertigo, diplopia, hemiplegia, nystagmus, Horner signs, ataxia	LMI, R+M, middle	VA stenosis	4 years	Pneumonia

*DM, diabetes milletus; HT, hypertension; CHD, coronary heart disease; ABO, atheromatous branch occlusion; AAE, artery-to-artery embolism; CE, cardiogenic embolism; SVD, small vessel disease; UN, undetermined etiology; DIS, dissection; LMI, lateral medullary infarction; MMI, medial medullary infarction; LMI+C, LMI+cerebellar infarction; MMI+C, MMI+cerebellar infarction; VA, vertebral artery; BA, basilar artery; VAH, vertebral artery hypoplasia; VBD, vertebrobasilar dolichoectasia; R, rostral; M, middle; C, caudal; V, ventral; D, dorsal*.

**Figure 3:**
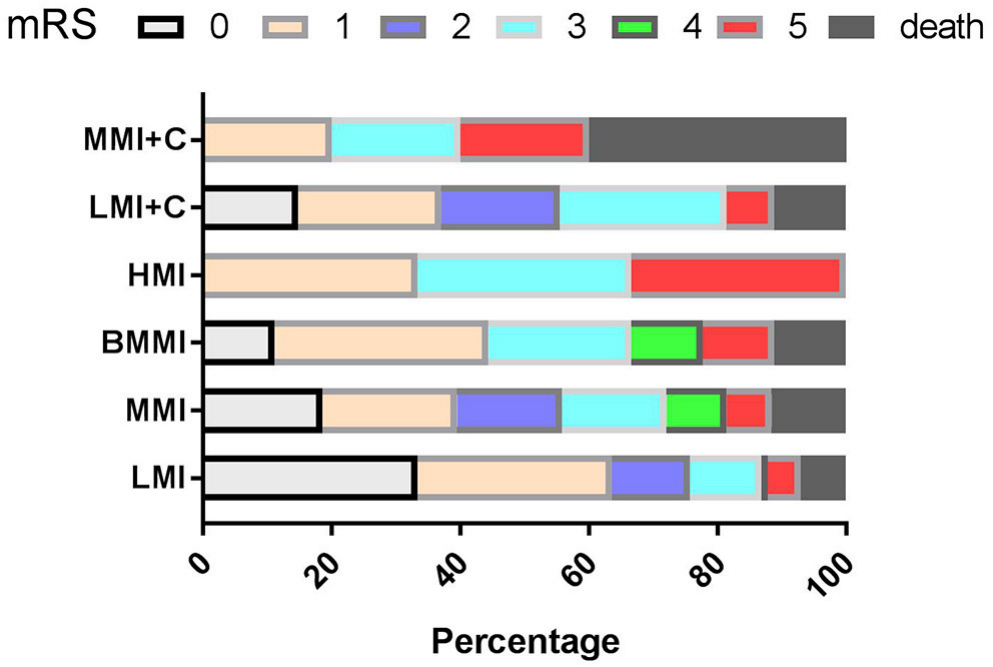
mRS according to the types of MI during follow-up. mRS, modified Rankin Scale; LMI, lateral MI; MMI, medial MI; LMI+C, LMI+cerebellar infarction; MMI+C, MMI+cerebellar infarction; BMMI, bilateral MI; HMI, hemimedullary infarction.

During follow-up, clinical outcomes were favorable in 122 patients and poor (MRS ≥ 3) in 38 (21.2%) patients (Figure 3). On multivariate analysis of baseline date at onset, age ≥ 65 years (OR = 5.306, 95%CI = 2.494–9.641, *P* < 0.001), dysphagia (OR = 3.909, 95%CI = 1.806–8.447, *P* < 0.001), and stroke recurrence (OR = 4.826, 95%CI = 1.348–17.914, *P* = 0.031) were found to be risk factors for poor prognosis. Multivariate analysis of the vertebrobasilar artery status of patients showed that > 50% BA stenosis (OR = 4.348, 95%CI = 0.102–0.932, *P* = 0.037) was a risk factor for poor prognosis. Multivariate analysis of different infarct sites showed that ventral MI (OR = 3.850, 95%CI = 0.219–0.879, *P* = 0.042) was a risk factor for poor prognosis.

**Figure 4:**
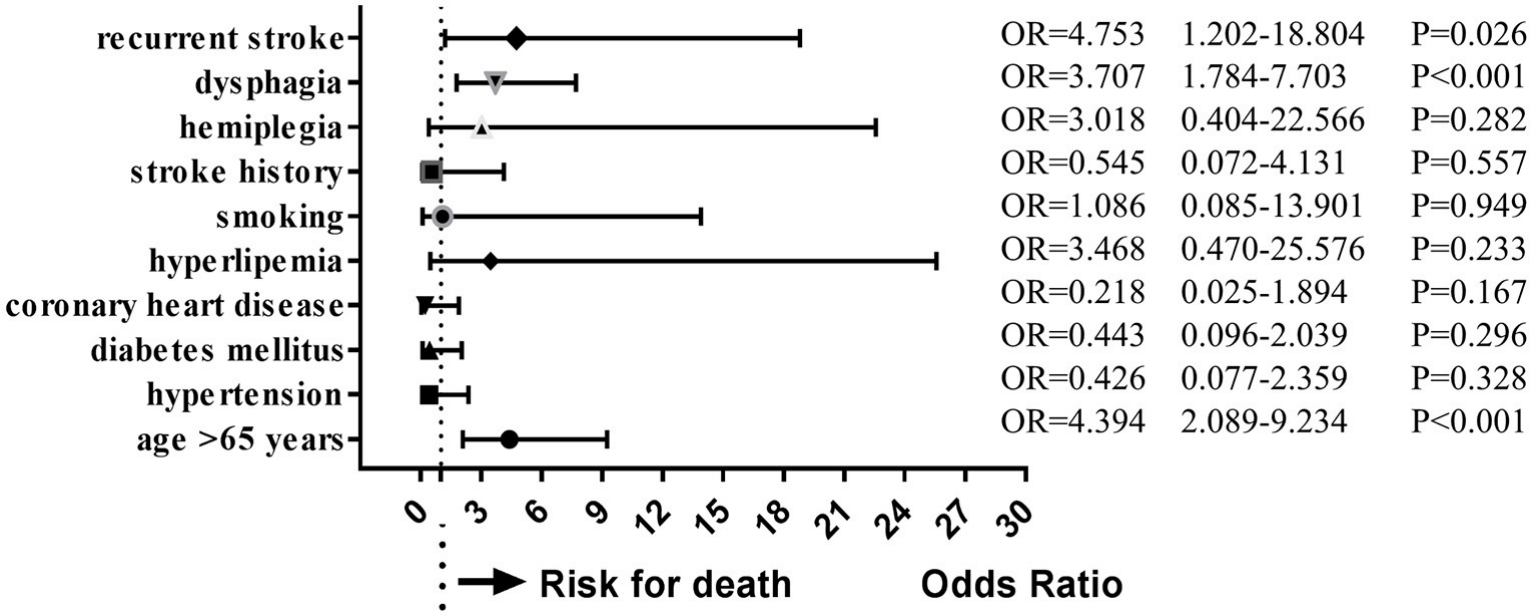
Clincal risk factors for death. Age ≥ 65 years, dysphagia, and stroke recurrence were risk factors for death.

**Figure 5:**
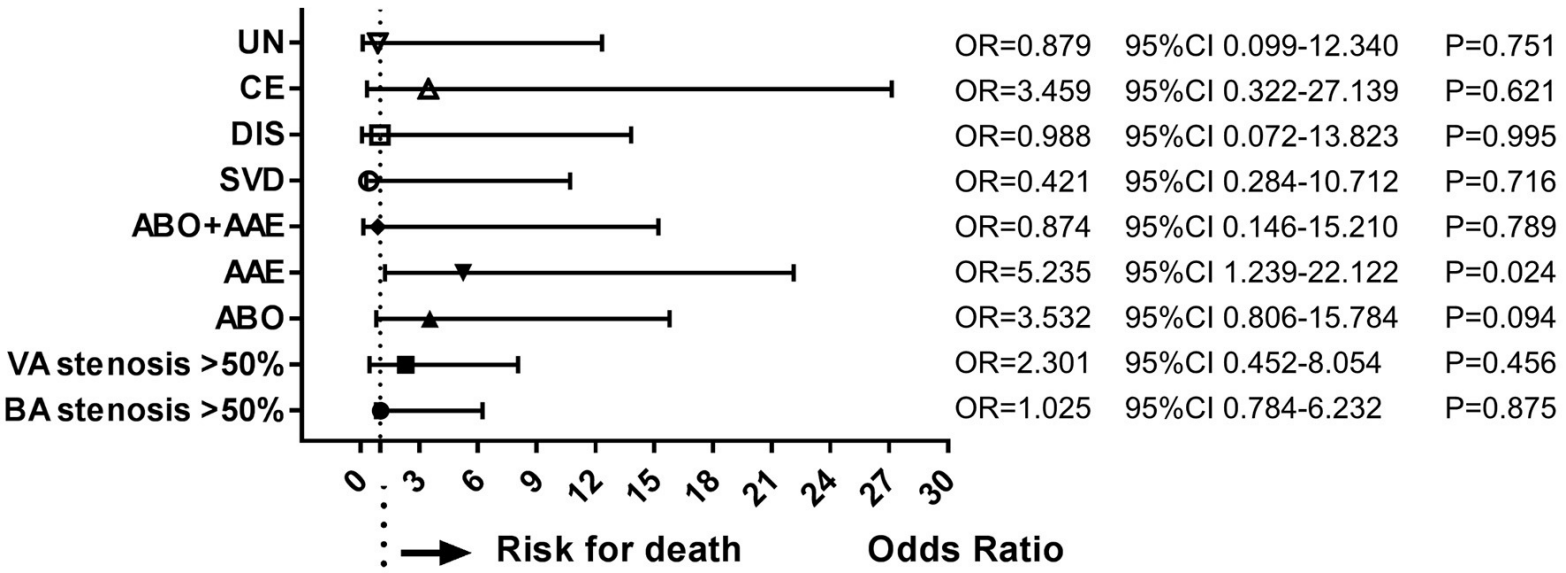
Risk factors of stroke mechanisms for death. AAE was a risk factor for long-term death. ABO, atheromatous branch occlusion; AAE, artery-to-artery embolism; CE, cardiogenic embolism; SVD, small vessel disease; DIS, dissection; UN, undetermined.

**Figure 6:**
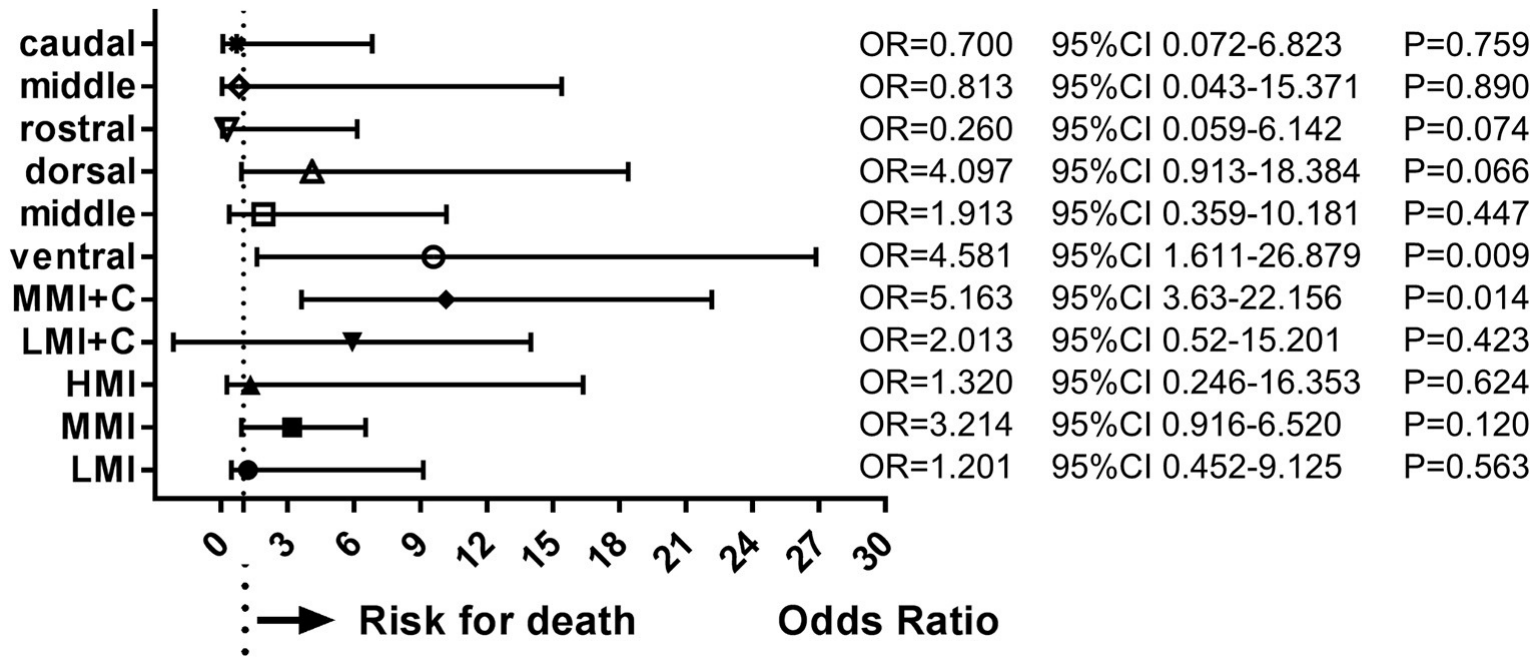
Risk factors of infarct sites for death. Ventral MI and MMI+C were risk factors for death. LMI, lateral medullary infarction; MMI, medial medullary infarction; LMI+C, LMI+cerebellar infarction; MMI+C, MMI+cerebellar infarction.

During follow-up, 19 patients (10.6%) had died. Multivariate analysis of baseline data at onset showed that age ≥ 65 years (OR = 4.394, 95%CI = 2.089–9.234, *P* < 0.001), dysphagia (OR = 3.707, 95%CI = 1.784–7.703, *P* < 0.001), and stroke recurrence (OR = 4.753, 95%CI = 1.202–18.804, *P* = 0.026) were risk factors for death (Figure 4). On multivariate analysis of the lesions of vertebrobasilar arteries and presumed stroke mechanism, AAE (OR = 5.235, 95%CI = 1.239–22.122, *P* = 0.024) was found to be a risk factor for death (Figure 5). Multivariate analysis of the different infarct sites showed that ventral MI (OR = 4.581, 95%CI = 1.611–26.879, *P* = 0.009) and MMI+C (OR = 5.163, 95%CI = 3.630–22.156, *P* = 0.014) were risk factors for death (Figure 6).

## Discussion

In this study, we compared the clinical symptoms, signs, and rostro-caudal and ventro-dorsal involvement in LMI, MMI, BMMI, LMI+C, and MMI+C based on a large cohort of MI patients involving a mean follow-up period of 42.7 months. This is in contrast to most previous studies that investigated the clinicotopographical correlation based on a dichotomized categorization of LMI and MMI. We also described the long-term prognosis of 179 patients with MI treated at the stroke centers of three tertiary hospitals. In the present study, 21.2% patients showed poor long-term prognosis (mRS ≥ 3) and 10.6% patients died. Eleven patients (6.1%) experienced recurrent stroke. In a study of long-term prognosis and mortality of all stroke patients at a Mexican hospital, 14.5% subjects died during the 4-year follow-up and the recurrence rate was 20.2% (14). The mortality and recurrence rates were higher than that in our series of medullary patients. A total of 20.9% of patients presented severe sequelae at hospital discharge, which was consistent with the present medullary case series. We further identified the risk factors for poor long-term prognosis and death using multivariate analysis.

With respect to the clinical features in our series, LMI syndrome (53.6%) was two times more common than MMI (23.5%). Interestingly, we also found three (1.7%) HMI (infarction area > 40% of the medulla in one place). Sensory impairment was the most common symptom and atheromatous branch occlusion was the most frequently presumed stroke etiology in the present study. On rostro-caudal classification, most lesions were located in the middle medulla (*n* = 65); on ventro-dorsal classification, most lesions were located in the ventral medulla (*n* = 32). These results could help us reconsider the clinical features of medullary infarction; however, this is consistent with a previous study (2).

In the present study, the most common characteristics of vertebrobasilar artery lesions were higher ipsilateral VAH or V4AH (42.5%) and ipsilateral VA stenosis >50% (34.6%), although some cases had coexisting ipsilateral V4 occlusion (12.3%) and VBD (17.9%). Hong et al. (15) assumed that unequal VA flow because of the asymmetric VAs is an important hemodynamic contributor to basilar artery curvature and development of peri-vertebrobasilar junctional infarcts. In this chronic process, VAH seemed to be the consequence of the interaction between the unequal VA flow and the basilar artery curvature, or just acted as an initial factor (16). A hypoplastic VA can also result in the ipsilateral occlusion of this vessel due to a direct decrease in blood flow and easy collapse of the vessel caused by the smaller intracranial VA caliber (17). A hypoplastic VA can cause ipsilateral posterior inferior cerebellar artery infarction by directly decreasing the blood flow in the smaller intracranial VA (18).

There is a paucity of studies on the long-term prognosis of patients with MI; in particular, there is no credible data on the prevalence of poor outcomes. In a recent study of 43 MI patients involving a median follow-up of 17 months, two patients died and two patients experienced recurrent ischemic events (19). A more recent investigation of 81 MI patients showed generally favorable outcomes (9). Based on these reports and other small series, MI has been regarded as a benign condition. However, the outcome is not uniformly favorable, especially for MMI patients. In a follow-up study of MMI, 16.2% (11/68) patients died (3). A systematic review of 38 BMMI patients revealed poor clinical outcomes with a 23.8% in-hospital mortality (20). In another study, 11.6% of LMI patients (5/43) died from respiratory and cardiovascular complications in the acute phase (5). In the present study, 21.2% patients showed poor long-term prognosis (mRS ≥ 3) and 10.6% patients died; our findings indicate the need for appropriate measures to improve the long-term outcomes of patients with MI. In a recent study of 248 LMI patients, 68 (36.1%) LMI patients showed poor functional outcome (mRS 2–6) at long-term follow-up (21); however, the authors could not determine the mortality rate.

A study by Caplan et al. revealed the poor prognosis of LMI patients more than 30 years ago (22). They reported two patients with LMI who experienced recurrent episodes of brainstem ischemia, and listed possible reasons for poor outcomes. However, the frequency and relative importance of the risk factors for long-term poor outcomes, including death, have not been fully studied in a large number of patients. Interestingly, in our study of 179 MI patients, age ≥ 65 years, dysphagia, and recurrent stroke were risk factors for poor prognosis and death. In a previous study of 157 LMI patients, older age, and initial dysphagia were found to be independent predictors of poor long-term prognosis (mRS ≥ 2) after acute LMI (23). In a study of 86 consecutive MMI patients, age and severe motor dysfunction at admission were predictors of poor prognosis (mRS > 3) (3). The association of old age and dysphagia with poor prognosis is likely explained by a higher prevalence of pneumonia in elderly and dysphagic patients as compared to that in their younger counterparts and those without dysphagia. Pneumonia is a major cause of death in stroke patients. In a study of recurrent ischemic stroke in a hospital-based population in Western Norway, age was independently associated with stroke recurrence and the recurrence significantly increased the all-cause mortality (24). In a study of all types of stroke involving the use of adjusted models, the prognostic factors for early and late survival after stroke were age and in-hospital medical complications (14). High comorbidity and recurrence increased the risk of late death but not the risk of early death (14).

Stroke-related mortality varies considerably between stroke types, regions, and countries (25). However, the correlates of long-term poor prognosis and death are not well characterized in the context of medullary infarction. We showed that BA stenosis >50% and ventral MI are risk factors for long-term poor prognosis; more importantly, ventral MI and MMI+C were potential risk factors for death. In a recent study of 248 LMI patients, LMI accompanied by ischemic lesions at other areas was associated with poor outcomes; on multivariate analysis, age, diabetes, presence of dysphagia, and pneumonia were independently associated with poor functional outcomes (21). Our findings suggested that not only LMI but also MMI accompanied by ischemic lesions in other areas were associated with poorer prognosis and death. In a study by Kim and Han (3), severe motor dysfunction suggestive of excessive corticospinal tract damage at admission was a predictor of poor prognosis; this is consistent with our findings that ventral MI and MMI+C usually cause motor dysfunction due to involvement of the pyramidal tract. In another study of 37 MI patients, more rostral lesion locations in LMI was correlated with a poorer 90-day outcome (mRS ≥ 2), while more dorsal lesion locations in MMI was correlated with a poorer 90-day outcome (6). This is probably because even without hemiplegia or quadriplegia, culprit lesions with persisting dysphagia affect the activities of daily living and increase the risk of pneumonia, which is a prognostic factor for MI.

In a study of 81 consecutive patients with acute isolated MI, large artery atherosclerotic occlusive disease and dissection compared with penetrating artery disease were independently correlated with poor outcome (mRS ≥ 2 and/or dysphagia) in LMI. Moreover, large artery atherosclerotic occlusive disease was significantly correlated with poor outcome in MMI (9). However, in our study, artery-to-artery embolism was a predictor of all-cause death during follow-up. Hyperintense plaques and a higher prevalence of plaque surface irregularity were more frequently observed in artery-to-artery embolism group by whole-brain high-resolution magnetic resonance imaging; this suggests that artery-to-artery embolic infarction is associated distinct vulnerable plaque characteristics (26). Early detection and treatment of rupture-prone vulnerable atherosclerotic plaques is critical to reduce mortality associated with cardiovascular disease (27). These findings are noteworthy, and further research is required to elucidate the exact relation between the presumed stroke mechanism and poor outcomes. Increasingly, novel imaging techniques have been applied to evaluate poor outcomes in MI patients. In a study of 34 MI patients, 18 had a normal perfusion status, while 16 had perfusion defects in the medulla and/or inferior cerebellum; on multivariate analysis, abnormal perfusion weighted imaging and DWI patterns were independently associated with poor early and late outcomes (mRS ≥ 3) following MI (28).

Some limitations of our study should be considered while interpreting the findings. First, arterial lesions were determined based on MRA, which is liable to show flow-related artifacts. Second, data pertaining to clinical features were retrospectively obtained from medical records. Third, there were a small number of patients with some types of MI (such as HMI and MMI+C) owing to their rarity; this may have introduced an element of bias. Fourth, the study excluded some important cases which may limit the applicability of our findings when discussing the prognosis with the caregivers of patients. Finally, the outcomes were assessed using the mRS. In fact, mRS is not an ideal tool to assess the outcomes of LMI. Although LMI patients may be categorized as having good outcomes based on mRS score, they often have severe sensory loss, central pain, or dizziness that are not reflected with mRS. However, currently, there are no better scales for assessment of long-term outcomes of stroke patients.

## Conclusions

In this study of a large cohort of MI patients, the clinical symptoms, signs, prognosis, and imaging findings of rostro-caudal and ventro-dorsal medulla were found to be different. A large proportion of patients showed poor long-term prognosis (21.2%), while the all-cause mortality rate was 10.6%. Age ≥ 65 years, dysphagia, recurrent stroke, and MMI+C were risk factors for poor prognosis and death.

## Data Availability Statement

The raw data supporting the conclusions of this article will be made available by the authors, without undue reservation.

## Ethics Statement

The studies involving human participants were reviewed and approved by the first affiliated hospital of Henan University of CM. The patients/participants provided their written informed consent to participate in this study. Written informed consent was obtained from the individual(s) for the publication of any potentially identifiable images or data included in this article.

## Author Contributions

DZ and SY researched literature and conceived the study. QM, MY, HZ, and XL were involved in protocol development, gaining ethical approval, patient recruitment, and data analysis. DZ and JZ wrote the first draft of the manuscript. All authors reviewed and edited the manuscript and approved the final version of the manuscript.

## Conflict of Interest

The authors declare that the research was conducted in the absence of any commercial or financial relationships that could be construed as a potential conflict of interest.

## Supplementary Material

The lost to follow up data as followed (25 patients): There were 13 males and 12 females, and the mean age was 63.2 ± 11.3. There were 11 lateral medullary infarctions (LMI), eight medial medullary infarctions (MMI), five lateral medullary infarction plus cerebellum infarctions (LMI+C), and one bilateral medial medullary infarction (BMMI).

## Abbreviations

LMI: lateral medullary infarction
MMI: medial medullary infarction
MI: medullary infarction
BMMI: bilateral medial medullary infarction
HMI: hemi-medullary infarction
LMI+C: LMI+cerebellar infarction
MMI+C: MMI+cerebellar infarction
VAH: vertebral artery hypoplasia
M: middle
D: dorsal
V: ventral
VA: vertebral artery
BA: basilar artery
LVD: large vessel disease
ABO: atheromatous branch occlusion
AAE: artery-to-artery embolism
CE: cardiogenic embolism
SVD: small vessel disease
DIS: dissection
UN: undetermined
mRS: modified Rankin scale.
