# Supplementary figures and images for: Risk Factors for Long-Term Death After Medullary Infarction: A Multicenter Follow-Up Study
Source: Front Neurol. 2021 Mar 4;12:615230. doi: 10.3389/fneur.2021.615230 (PMC7969705; doi:10.3389/fneur.2021.615230)

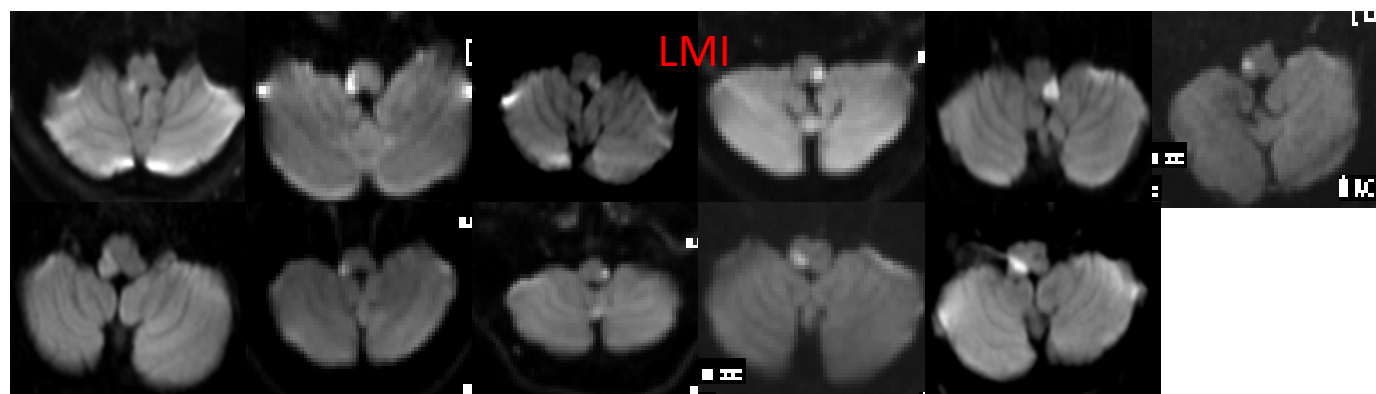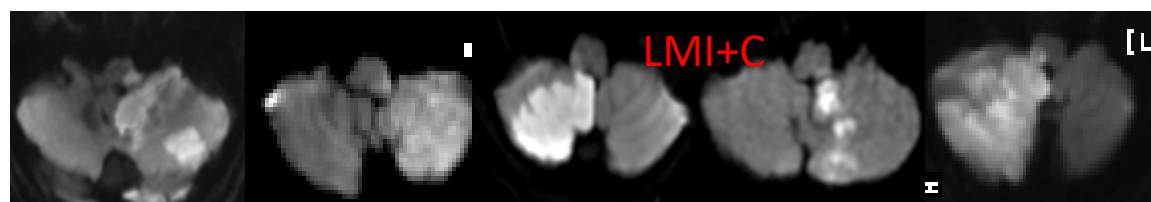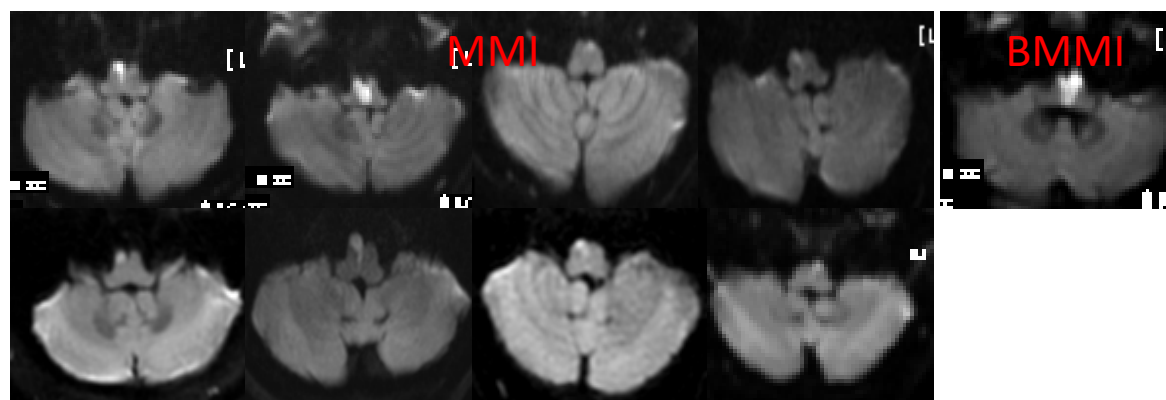

Supplement: Supplementary file 1 [file Image_1.pdf]
