# Supplementary material for: Risk Factors for Long-Term Death After Medullary Infarction: A Multicenter Follow-Up Study
Source: Front Neurol. 2021 Mar 4;12:615230. doi: 10.3389/fneur.2021.615230 (PMC7969705; doi:10.3389/fneur.2021.615230)
Supplement: Supplementary file 2 [file Table_1.pdf]

Table 1 Charactersitics of lost to follow up 25 patients with medullary infarction

Patient	Age/sex	Risk factors	Stroke mechanisms	Major symptoms and signs	Lesions location of medulla	Magnetic resonance angiography
1	80/M	HT, drinking	ABO	vertigo ,vomit, hemiplegia,dysphasia	MMI,rostral,ventral	VA stenosis
2	42/M	smoking	UN	vertigo, hemidysesthesia, dysphasia	LMI, middle, dorsal	V4AH
3	65/M	HT,DM,smoking	ABO	vertigo,hemidysesthesia,hemiplegia,dysphasia	MMI,rostral,ventral	BA and VA stenosis
4	50/M	HT,DM,drinking	UN	vertigo, vomit, hemidysesthesia,dysphasia	LMI,middle, dorsal	V4AH
5	47/F	HT,DM	AAE	vertigo, hemiplegia	MMI, middle,M+D	BA stenosis
6	71/F	HT,DM ,stroke	UN	vertigo,ataxia,dysarthria, hemidysesthesia,dysphasia	LMI,caudal, middle	V4AH
7	69/M	HT,DM,CHD,stroke,hyperlipemia	AAE	vertigo,facial paralysis, dysarthria, nystagmus, hemiplegia,dysphasia	LMI+C,R+M,dorsal	VBD
8	74/M	HT,smoking,drinking	ABO+AAE	vertigo,ataxia,nystagmus, hemidysesthesia	LMI+C, middle, dorsal	VA stenosis
9	79/F	HT,DM	DIS	hemidysesthesia, hemiplegia	LMI,middle, ventral	VA string
10	68/M	CHD,stroke,smoking,drinking	UN	vertigo,vomit, monoplegia, dysarthria, hemidysesthesia,nystagmus	LMI,R+M, dorsal	V4AH
11	59/F	DM	AAE	vertigo,hemiplegia,dysphasia	MMI,R+M, ventral	VBD,VAstenosis
12	77/M	HT, atrial fibrillation,stroke	CE	dysarthria,facial paralysis, quadriplegia,dysphasia	BMMI,rostral,ventral	BA stenosis
13	65/F	HT, CHD,stroke,hyperlipemia	ABO+AAE	vertigo,vomit,dysarthria,hemidysesthesia,nystagmus,	LMI,R+M,M+D	VA stenosis
14	71/F	DM, hyperlipemia	UN	vertigo,dysarthria,nystagmus, ataxia,dysphasia	LMI,M+C,middle	V4AH
15	68/F	HT, CHD	AAE	vertigo,facial paralysis, ataxia, hemidysesthesia, nystagmus, hemiplegia,dysphasia	MMI,middle,ventral	VA stenosis
16	49/F	HT,CHD, atrial fibrillation	CE	vertigo,facial paralysis,ataxia, hemidysesthesia, hemiplegia	MMI,rostral,V+M+D	VA stenosis
17	58/F	HT	ABO	vertigo,vomit,dysphasia,hemidysesthesia,nystagmus, hemiplegia	MMI,middle,M+D	VA stenosis,VAH

18	83/M	HT,stroke,smoking	SVD	vertigo,hemidysesthesia,hemiplegia,ataxia	LMI,rostral,dorsal	normal
19	66/F	HT,DM,CHD,atrial fibrillation	CE	vertigo,hemiplegia, nystagmus,Horner signs,ataxia	LMI+C,R+M,middle	VA stenosis
20	49/M	DM,CHD,smoking	UN	vertigo,hemiplegia, nystagmus, dysphasia,ataxia	LMI+C,M,caudal	VBD
21	58/M	DM,HT,smoking	SVD	vertigo,hemiplegia, nystagmus	LMI,rostal,middle	VA stenosis
22	44/M	HT,smoking	DIS	vertigo, nystagmus, dysphasia,ataxia	LMI, rostral,dorsal	VA string
23	68/F	DM, HT,stroke	ABO+AAE	headache, hemiplegia,dysphasia	MMI,middle-caudal,V+M+D	VA stenosis
24	70/F	HT,DM,CHD	AAE	vertigo, nystagmus, dysphasia,ataxia	LMI+C,R,dorsal	VA stenosis
25	50/M	HT, hyperlipemia	SVD	vertigo, nystagmus	LMI, rostal, caudal	V4AH

DM, diabetes milletus; HT, hypertension; CHD, coronary heart disease; ABO, atheromatous branch occlusion; AAE, artery-to-artery embolism; CE, cardiogenic embolism; SVD, small vessel disease; UN, undetermined etiology; DIS, dissection; LMI, lateral medullary infarction; MMI, medial medullary infarction; LMI+C, LMI+cerebellar infarction; MMI+C, MMI+cerebellar infarction; VA, vertebral artery; BA, basilar artery; VAH, vertebral artery hypoplasia; VBD, vertebrobasilar dolichoectasia; R, rostral; M, middle; C, caudal; V, ventral; D, dorsal
